# “*I Felt Empowered*”: Patient-Reported Experience with a Pilot National Community Pharmacy-Based Urinary Tract Infection Service

**DOI:** 10.3390/antibiotics14111086

**Published:** 2025-10-28

**Authors:** Efi Mantzourani, Andrew Evans, Rhian Deslandes, Haroon Ahmed, Nicola Reeve, Samuel Macdonald, Rebecca Cannings-John

**Affiliations:** 1 Cardiff School of Pharmacy and Pharmaceutical Sciences, Cardiff University, King Edward VII Avenue, Cardiff CF10 3NB, UK; deslandesre@cardiff.ac.uk; 2 Digital Health and Care Wales, NHS Wales, Cardiff CF11 9AD, UK; 3 Primary Care Services, Welsh Government, Cardiff CF10 3NQ, UK; andrew.evans@gov.wales (A.E.);; 4 Division of Population Medicine, Cardiff University, Cardiff CF14 4YS, UKreeven1@cardiff.ac.uk (N.R.); 5 Centre for Trials Research, Cardiff University, Cardiff CF14 4YS, UK

**Keywords:** community pharmacy, pharmacy services, Choose Pharmacy, urinary tract infections, UTI, antimicrobial stewardship, diagnostics, PREMs, patient reported experience measures, patient satisfaction, patient education, antibiotics, Common Ailments Service

## Abstract

**Background:** In June 2024, urinary tract infections (UTIs) were added to the list of conditions that could be treated by community pharmacists providing the national Common Ailments Service in Wales. The aim of this study was to describe patient-reported experiences of UTI management by pharmacists. **Methods:** A positivist research paradigm was selected, with data collection through a survey. **Results:** In total, 309 surveys were received between 29 June 2024 and 14 July 2025. Patients rated their experience using a scale of 1 (very poor) to 10 (excellent), with a median score of 10 (IQR = 10 to 10, range 6 to 10). High satisfaction was independent of age and provision of antibiotics, with the same median and IQR and a similar range between the groups who received and did not receive antibiotics (7–10 and 6–10, respectively). Of the 309 respondents, 297 (96.1%) stated that the next time they had a UTI, they would return to the pharmacy instead of trying to see a GP, and 253 (81.9%) that they understood why antibiotics are not always recommended. **Conclusions**: Community pharmacists managed patient expectations, improved patient confidence in managing current symptoms and provided information on self-care strategies for preventing future infections, demonstrating their valuable role in health promotion and antimicrobial stewardship.

## 1. Introduction

Urinary Tract Infection (UTI) is usually caused by bacteria from the gastrointestinal tract entering the urinary tract, from which it spreads to the bladder. It is one of the most common presentations in primary care [1], accounting for 1–3% of GP appointments [2]. Symptoms include dysuria, urinary frequency and urgency. Appropriate and timely access to care can help to avoid complications

The National Health Service (NHS) is a state-funded healthcare system providing universal medical services to UK residents. Wales is a semi-autonomous region of the UK with devolved powers over health and the operation of the NHS within its area. Healthcare is delivered through NHS hospitals providing secondary and tertiary services and independent primary care contractors (e.g., general practitioners (GPs) and community pharmacists) commissioned by seven health boards.

For most patients, primary care is the first point of access for new or ongoing symptoms. Internationally, the role of community pharmacists has evolved significantly over time, moving from being focussed on the supply of medicines to the provision of clinical services and patient-centred care; this coincides with policies which move to more care being provided in the community.

In Wales, government and pharmacy bodies have committed to maximise the use of community pharmacists’ skills and have, over a number of years, increased the range of clinical services provided by pharmacies [3]. There are approximately 700 community pharmacies in Wales, which, in general, are easily accessible and able to support patients with their care.

The Common Ailments Service (CAS), initiated in 2013 is provided by almost all pharmacies in Wales and enables pharmacists to see and treat 27 common conditions at no charge to service users [4]. The CAS allows pharmacists to supply some prescription-only medicines under Patient Group Directions (PGD) and aims to reduce pressure on general practices. In June 2024, urinary tract infections (UTIs) were added to the list of conditions that could be treated by pharmacists, following a successful pilot in one health board area [5].

The service allows women aged between 16 and 65 with uncomplicated UTI to have their symptoms assessed, undergo point of care urinalysis in line with national guidance [6], and be treated with antibiotics where clinically indicated [7]. Women over 65, women who are pregnant, men and women with specified co-morbidities are not eligible for treatment. An important element of UTI management for community pharmacists is to support appropriate antimicrobial stewardship, a role acknowledged by the Royal Pharmaceutical Society, the professional leadership body for pharmacists in Great Britain [8]. Pharmacy UTI consultations are recorded in *Choose Pharmacy*, a digital platform which provides access to a patient’s medication history, enables information to be shared by the pharmacy with a patient’s GP, and calculates pharmacy remuneration.

Uncomplicated UTIs are treated by community pharmacists in all other parts of the UK, through nationally commissioned NHS services in Scotland [9], England [10] and Northern Ireland [11]. Previous studies found that patients in Scotland were satisfied with the service, had been seen in a timely manner and, should it not have been available in community pharmacy, they would otherwise have attended the GP or emergency services [12]. Stewart et al. (2018) [13] also reported high levels of patient satisfaction, highlighting trust in pharmacists, with clinically appropriate treatment being provided. In England, a pilot study of a ‘paid for’ community pharmacy UTI service found that. of those who received the service. approximately 71% would have otherwise attended their general practice [14]. These studies demonstrate how community pharmacy UTI services could result in a reduction in consultations in other healthcare settings. Peiffer-Smadja et al. [15] and Lee et al. [16] have illustrated how pharmacists in the community can play a more significant role in treating UTIs in England.

Internationally, UTIs are managed in community pharmacy in a number of countries and this has received growing attention over recent years [17]. This includes Australia [18], New Zealand [19], Switzerland [20], The United States [21] and Canada [22]. The authorisation for the pharmacist to prescribe and the medication supply route differ between countries; however, common themes of patient acceptability and safe care are observed. Nissen et al. illustrated how patients in Australia valued pharmacists treating their UTI, providing positive views, especially around the convenience of access to care [18]. Beahm et al. investigated the outcomes of UTI management by pharmacists and patient satisfaction in Canada [22]. They discovered that clinical cure was seen in 88.9% of patients, with patients reporting trust in the pharmacist, that they valued the increased accessibility and would recommend the service to others. A systematic review conducted by Swart et al. (2024) showed how management by pharmacists was a cost-effective option when compared to GP management [23].

There is a considerable body of literature demonstrating that services offered in community pharmacy are generally well received by the public [24,25,26,27,28]. Many papers report patient satisfaction; however, patient-reported experience measures (PREMs) allow researchers to explore patient experience of a particular interaction rather than purely exploring their satisfaction. PREMs are commonly used in healthcare evaluations and assist in improving service provision [29]. They are deemed to be more comprehensive and allow patients to report their experience objectively via tools such as surveys, which is critical to improve pharmacy care.

The aim of this study was therefore to describe patient-reported experience with the pilot national community pharmacy-based UTI service in Wales.

## 2. Results

In total, 309 completed surveys were received between 29 June 2024 and 14 July 2025 (52 paper and 257 electronic surveys; English n = 308, Welsh n = 1). Whilst a response rate cannot be calculated as the number of patients given the opportunity to complete the survey is unknown, the number of participants was a small sample of the total population who presented to the pharmacy for a UTI consultation (n = 9077 between 17 June 2024 and 31 January 2025).

The majority of patients responding to the survey were between the ages of 18–24 years (n = 70, 22.7%) with 20.4% (n = 63) aged 35–44 and 20.4% (n = 63) aged 45–54 years. Of the 309 who responded, 140 (45.3%) previously tried to see a GP about their UTI symptoms, 242 (78.3%) expected that they might be able to receive an antibiotic from the pharmacy, and 234 (75.7%) stated that they were supplied an antibiotic from the pharmacy. These characteristics are broadly similar to characteristics from data in relation to the 9077 consultations for the UTI service between 17 June 2024 and 31 January 2025 (Supplementary Table S2).

### 2.1. Patient Experience with the Service and Its Delivery

Patients rated their experience using a scale of 1–10, where 1 was very poor and 10 was excellent; 278 (90.0%) scored the service a 10, 20 (6.5%) scored it a 9, and 9 (2.9%) scored it an 8 (median score = 10, IQR = 10 to 10, range 6 to 10). When examined by whether the patient reported receiving an antibiotic from the pharmacy, both groups rated their experience with the service highly (antibiotic supplied: median score 10, IQR 10 to 10, range 7 to 10; antibiotics not supplied: median score 10, IQR 10 to 10, range 6–10). When tested, no differences were found in patient experience scores between age-groups (*p*-value = 0.317) and antibiotic supply (*p*-value = 0.051).

**Figure 1:**
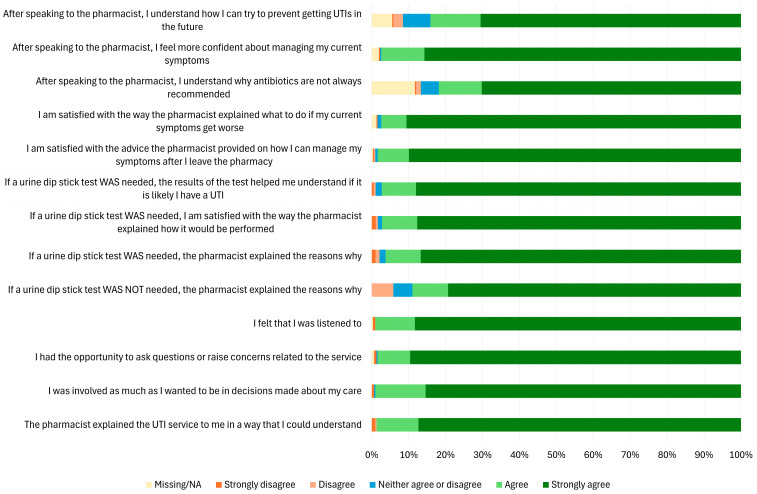
A bar chart showing the percentage patient experience in relation to different aspects of the urinary tract infections (UTI) service and its delivery.

Figure 1 presents a breakdown of patient experience with different elements of the service and its delivery (full data on survey responses is included in Supplementary Table S1). Overall, 305/309 (98.7%, 95% CI: 96.7% to 99.5%) patients either agreed (n = 35, 11.3%) or strongly agreed (n = 270, 87.4%) that the pharmacist explained the urinary tract infection service in a way that was understood and 306/309 (99.0%, 95% CI: 97.2% to 99.7%) patients agreed or strongly agreed that they were involved as much as they wanted to be in decisions made about their care, that they had the opportunity to ask questions or raise concerns related to the service and that they felt listened to.

The number of patients responding to whether a urine dip stick test was and was not needed did not add up to the total number of surveys, as some patients responded positively to both questions, and some responded to neither. Of the 155 patients who reported not needing a urine dip stick test, 138 (89.0%) agreed that the pharmacist explained the reasons why it was not needed, and 9 (2.9%) disagreed. Where a patient stated that a urine dip stick test was needed, 182/205 (88.8%) agreed or strongly agreed that the pharmacists explained the reason why, 174/179 (97.2%) were satisfied with the way the pharmacist explained how it would be performed, and 179/184 (97.3%) agreed or strongly agreed that the results of the test helped them understand if it was likely they have a UTI.

All except four patients were satisfied with the advice the pharmacist provided on how current symptoms could be managed after leaving the pharmacy, and with the way the pharmacist explained what to do if symptoms got worse. Regardless of whether or not patients needed a urine sample, 301 (97.5%) felt more confident about managing their current symptoms and 260 (84.2%) understood how they could prevent getting UTIs in the future after speaking to the pharmacist. A total of 253 patients (81.9%) agreed or strongly agreed that after speaking to the pharmacist they understood why antibiotics are not always recommended; 5 (1.6%) disagreed or strongly disagreed; 15 neither agreed nor disagreed; and 36 (11.6%) stated that this information was not applicable (n = 35) or was missing (n = 1) (potentially indicating that this was not discussed).

### 2.2. Health-Seeking Behaviour

All bar one patient (n = 308, 99.7%) agreed that they would recommend the pharmacy UTI service to others; the one patient that strongly disagreed, scored their experience with the service a 10 but stated that they “*had to visit 3 pharmacies before I came across one that offer [sic] me the help that I needed*”. Of the 309 respondents, 297 (96.1% 95% CI 93.3% to 97.8%) agreed or strongly agreed that the next time they had a UTI, they would return to the pharmacy instead of trying to see the GP; 9 (2.9%) were not sure (neither agree or disagree) and 3 (1.0%) disagreed/strongly disagreed. 

### 2.3. Free-Text Comments

**Figure 2:**
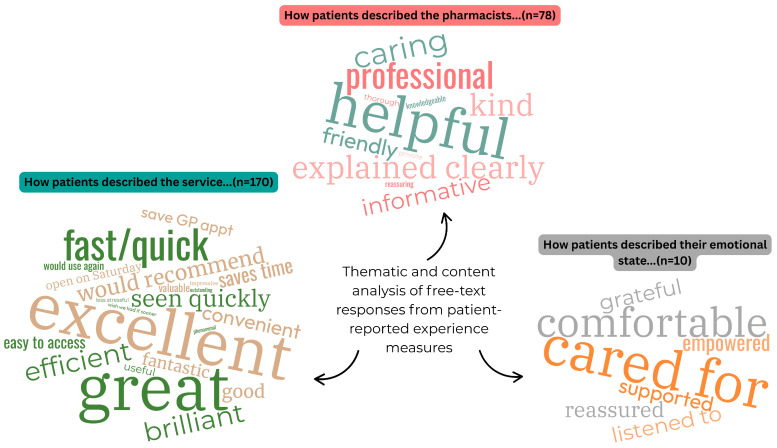
Word cloud based on the content analysis of free-text comments (n = 149) in the national community pharmacy pilot UTI service patient experience surveys, presented by theme.

A total of 149 (48.2%) patients added a free-text comment to the survey. Inductive analysis allowed us to construct three themes: how patients described the service; the pharmacists; their emotional state. Content analysis in relation to how patients described the service returned a total of 170 occurrences of terms such as “phenomenal/outstanding/excellent/good/great” (n = 86), “fast/quick” (n = 14), “efficient” (n = 13), “convenient” (n = 10). Content analysis in relation to how patients described the pharmacists returned a total of 78 occurrences of terms such as “helpful” (n = 23), “kind/caring/lovely/friendly” (n = 16), “explained clearly/well/through” (n = 14), “professional” (n = 9), “informative” (n = 6). Lastly, content analysis in relation to how patients described their emotional state returned a total of 10 occurrences of terms such as “comfortable” (n = 5), “listened to” (n = 2) and “supported”, “empowered” and “grateful” (all n = 1). Table 1 summarises representative quotes from each theme; Figure 2 summarises the content analysis in word clouds for each theme. A total of eight negative comments were left, and these related to lack of service availability in all pharmacies (n = 2); staff not yet being familiar with service delivery requirements (n = 2); inappropriate referral to service by GP staff (n = 1); patient not meeting inclusion criteria (n = 1); patient perception that service inclusion criteria too restrictive (n = 1); and patient not knowing in advance that a urine sample may be needed (n = 1).

## 3. Discussion

This is the first study reporting on systematically collected PREMs from an NHS-funded national community pharmacy UTI service in the UK. We analysed patient experience survey data to evaluate patient acceptability and assess whether the service had an impact on participants’ intentions to prioritise community pharmacists over GPs for future UTI care, in line with the broader policy shift of moving care closer to home [30].

An analysis of patient data from the initial thirteen months of the UTI service revealed high patient acceptability, with a median satisfaction score of 10, adding to the body of evidence reported in the literature from pharmacy services nationally [9,10,11,12,13,14,15,16] and internationally [17,18,19,20,21,22]. Free-text comments found that some of the key reasons related to the convenience of receiving the service in an accessible setting, efficiency of the service, and professionalism of the pharmacist and wider team, with comments sharing emotional states of relief for being able to access care outside of routine GP opening hours. This was reflected in the 96% of patients who agreed/strongly agreed that they will present to a community pharmacy the next time they have symptoms of a UTI, indicating a self-reported health-seeking behaviour change from the initial intentions whereby almost half of the patients reported having tried to see a GP for their symptoms. Redirecting the management of uncomplicated conditions from general practice settings to community pharmacies is a key aim for governments internationally [17], and in both the NHS England Long Term Plan [31] and the Welsh Government’s vision for ‘A Healthier Wales’ [30]. Between 17 June 2024 and 31 January 2025, 9077 consultations were recorded in *Choose Pharmacy* as being managed by the pharmacist via the service, highlighting the potential for considerable savings in GP appointments if the management of uncomplicated UTI can be transferred to community pharmacy. It is acknowledged that UTIs are self-limiting and PGD-based services risk overuse of antibiotics. However, the service’s clear protocol, use of urinalysis POCT and sharing of records between pharmacies and general practice, as suggested by Peiffer-Smadja et al. [15], should together minimise instances of overtreatment.

Satisfaction was found to be independent of age and the provision of antibiotic supply, with the same median and IQR between the patient group who received antibiotics and those who did not, and a very similar range (7–10 and 6–10, respectively). Lecky et al. found that most GPs reported that women expected antibiotic therapy for their UTI, but also suggested that when patients seek advice from a healthcare professional for managing UTI symptoms, their objective is not only to obtain an antibiotic prescription, but also for reassurance and a sense of control over their symptoms [32]. Results show that 97.5% of participants felt empowered in terms of managing their current symptoms, and this could explain the similarity in satisfaction scores between the two patient groups, demonstrating the pharmacist’s contribution in managing patient antibiotic expectations.

The highly similar satisfaction levels, coupled with the 81.9% of patients reporting that they understood why antibiotics are not always recommended after receiving information around antimicrobial stewardship (AMS), reaffirm the value of pharmacists in optimising antimicrobial use and improving patient outcomes. This finding aligns with previous research that has suggested community pharmacists were confident as the first professional contact for service users with uncomplicated UTI [15] and could play a greater role in its management [33]. The structured service in Wales provided time and access to medical information, identified by Jones et al. [34] as the main reasons preventing pharmacy staff giving self-care advice about UTI, and led to more than 84% of patients in the current study stating that they understand how to prevent getting UTIs in the future.

A small number of challenges were reported by patients, with the main one relating to inconsistent service availability and provision, a complex issue with multiple interconnected root causes, widely acknowledged in the literature. Financial pressures and the perceived unsustainable contractual framework for pharmacies [35], workforce shortages and workload causing burnout [36], geographical disparities and declining availability of community pharmacies [37] have all been cited as contributing factors in some areas in the UK. A step-by-step approach in service development, including piloting before wider implementation, and an increase in the community pharmacy contractual framework [38], may be pivotal in preparing the workforce and ensuring integration of clinical services within national contracts, as exemplified by the Sore Throat Test and Treat service in Wales [39].

### Strengths and Limitations

This was a nation-wide study, covering all health boards, and all eligible pharmacies were asked to promote the survey to patients consulting with the service, irrespective of the outcome of the consultation. Even though the survey’s distribution by pharmacists could have introduced response bias and influenced patient responses, we tried to minimise this by adopting a hybrid approach with electronic completion of the survey encouraged and pre-paid envelopes provided for paper copies, ensuring responses were shared directly with the research team, and by including in the service specification that pharmacists signed up for that all patients that were eligible for the service should be invited to take part in the study. The survey had a small number of items to improve completion rates, and we did not test for Cronbach’s alpha, which may impact the survey’s internal consistency and overall reliability. The survey only included one open-ended question, which limits qualitative depth. It is acknowledged that the sample size represents only a small sample of patients presenting for treatment, but we found the rate of antibiotic supply and demographic data to be broadly comparable, and as such, it can be assumed as representing the total treated patient group. Future health-seeking behaviour is patient self-reported and needs to be validated with a longitudinal study by following patients across their NHS journey. Nevertheless, these initial results are an indication of the service’s potential success in shifting health-seeking behaviour. This study was part of a larger evaluation of the UTI service and findings need to be interpreted within this context. A different study investigated data derived from the *Choose Pharmacy* platform, reporting on service outcomes and patient-reported outcome measures, including antibiotic use arising from the pharmacy and any additional healthcare consultations patients may have needed. Results from both studies were presented to policy-makers and have influenced the decision to make the service an integral part of the national Clinical Community Pharmacy Service from October 2025 [39]. Future longitudinal studies can explore reduction in inappropriate antibiotic use.

## 4. Methodology

### 4.1. Service Development and Implementation

A consultation template was developed in *Choose Pharmacy*, enabling pharmacists to provide and collect data about UTI service consultations. Patients were eligible for the service if aged between 16 and 65 years and if they met the clinical criteria described in the monograph developed by the Welsh Medicines Advice Service and approved by the All Wales Medicines Strategy Group (service specification) [7].

The diagnostic pathway, including requirements for urine sampling and a urinalysis point-of-care diagnostic test (POCT), was based on the current guidelines for diagnosis and treatment of acute uncomplicated UTI from the National Institute for Health and Care Excellence (NICE) and the UK Health Security Agency (UKHSA) [6]. The service could be accessed by women or transgender men who had not undergone sex reassignment surgery, aged between 16 and 65 years, who were not pregnant or catheterised. For patients meeting the criteria, a PGD allowed the supply of prescription-only medicines, including antibiotics (Supplementary Figure S1). Community pharmacies in Wales already commissioned to provide the CAS [39] were eligible to be commissioned to provide the UTI service upon completion of the required training [40]. The new service was commissioned on 17 June 2024, and 49 pharmacies across all health boards in Wales participated in the pilot.

### 4.2. Study Design

As the aim was to objectively describe a range of patient experience factors from as large a patient population as possible, a positivist research paradigm was selected [41], with a quantitative methodology. The study was based on primary data collection with a survey as a data collection tool.

### 4.3. Data Collection Tool

The survey was designed in collaboration with service commissioners, pharmacy representatives, public health professionals, experts in value-based healthcare and members of the public. Its development was based on a combination of literature review, including the national PREMs, and stakeholder-informed phrasing of questions around the objectives of the study:

- Describing experience with shared decision-making.
- Information pharmacists provided about the service and the role of urinalysis point-of-care testing.
- Advice pharmacists provided on managing current symptoms, safety netting and preventing future infections.
- Level of information pharmacists provided in relation to antimicrobial stewardship.
- Reported future healthcare behaviour.

The survey included the following: closed questions in relation to the service, for which patients had to state their level of agreement based on a 5-point Likert scale (1: strongly disagree, 5: strongly agree); patient demographics; action taken before coming to the pharmacy; future intentions in relation to seeking help from a healthcare professional for UTI symptoms; and an open-ended question available at the end of the survey to allow patients the opportunity to explain answers and provide information that may help support the quantitative data collected (Supplementary Table S1). No identifiable patient details were collected as part of the study. The survey responses were completely anonymous, and there was no way of linking any of the information provided back to participants.

An electronic version of the survey was created using Online Surveys^®^ v3 and piloted with members of the public (n = 16), testing face and content validity. The average completion time was less than 4 min, and all participants reported the language in the questions was easily understood. Some suggestions were provided and actioned, including creating a separate section for future actions, and including questions about preventing future infections.

### 4.4. Population and Sampling

As part of community pharmacists signing up to provide the service, they agreed to take part in this evaluation. The service specification outlined that no sampling of patients would be carried out. Instead, a census approach was adopted, and pharmacists had to invite all patients who had a UTI consultation at a pharmacy to take part in the survey, minimising response and social desirability bias. If a patient lacked capacity and was accompanied by a carer, the carer was asked to complete the survey on the patient’s behalf.

### 4.5. Recruitment

A sample size of 270 was estimated for statistical power to be reached. This was calculated using G*Power 3.1.9.7, and based on the 6 age groups, 90% power, 5% Type I error, and a difference in average experience rating between groups of 0.25. Data continued to be collected until the sample size was reached.

A hybrid approach was adopted for recruitment. Two sets of laminated participant information sheets (PIS) (in English and Welsh) were available in each participating pharmacy, one in the consultation room and one on the healthcare counter. The PIS had a QR code, which patients/carers could scan to complete the survey on their own electronic devices in the pharmacy, or photograph and complete later. Hard copies of the survey (in English and Welsh) were also available in the pharmacy and offered to patients/carers who preferred to complete the survey this way. A pre-paid envelope was provided for the return of those surveys. Patients were asked at the start of the survey to confirm that they had read the information sheet and that they understood that the return of a completed electronic or paper survey implied consent. Raw data from the electronic surveys and paper surveys were sent directly to the study team, ensuring that confidentiality was maintained and minimising social desirability bias. Data from any paper surveys were entered on Online Surveys^®^ v3 within a week of receipt.

### 4.6. Data Analysis

This was a descriptive study with objectives to describe:

- The patient experience of shared decision-making in the service.
- The patient experience of information pharmacists provided about the service and the role of urinalysis point-of-care testing.
- The patient experience of advice pharmacists provided on managing current symptoms, safety netting and preventing future infection.
- The level of information pharmacists provided in relation to antimicrobial stewardship.
- The impact of patient experience on reported future healthcare behaviour.

When sample size was reached, data from both the Welsh and English versions were extracted from Online Surveys^®^ v3 and exported to Excel^®^ Microsoft 365, with a 10% validation check to quality assure the data transfer. Data from the Welsh version were translated, where appropriate, and incorporated in a master dataset. The dataset was coded and transferred to IBM SPSS v29 [42], where descriptive analyses, such as frequencies alongside percentages, and measures of central tendency (mean, median) and variance (standard deviation, interquartile range (IQR) and range), were used to summarise the data. Ninety-five percent confidence intervals (CIs) were used to show precision for key proportions such as satisfaction and intention to use the service. To test for differences in the distribution of the patient experience score between age groups and antibiotic supply, an independent-samples Kruskal–Wallis and Mann–Whitney Test was used, respectively. To explore the representativeness of the sample in the study against the total population cohort who had a UTI consultation, consultation data were obtained from the *Choose Pharmacy* platform between 17 June 2024 and 31 January 2025. Free-text comments were coded in Microsoft Excel^®^ v2207 and a combination of content and thematic analysis was undertaken. Even though a Cohen’s kappa was not calculated, thematic coding was independently carried out by a second researcher, and no differences were found.

### 4.7. Ethical and Regulatory Considerations

The study protocol and all study documentation, including the cover letter for pharmacists, participant information sheet (in English and Welsh), and survey (in English and Welsh), were reviewed by the Cardiff School of Pharmacy and Pharmaceutical Sciences Research Ethics Committee and the study obtained approval in May 2024 (ref: 2324-20).

## 5. Conclusions

This first ever study reporting on PREMs collected directly from patients consulting with an NHS-funded national community pharmacy UTI service in the UK revealed high satisfaction levels that were independent of age and supply of antibiotics. Given the likely high expectation of antibiotic supply, the study indicates community pharmacists are able to manage patient expectations, improve patient confidence in managing current symptoms and provide information on self-care strategies for preventing future infections, exemplifying their role in healthcare education for AMS. Further mixed methods and longitudinal evaluation studies will add to this initial exploratory evidence.

## Figures and Tables

**Table 1:** Themes, and representative quotes as constructed by inductive thematic analysis of the free-text comments (n = 149) in the national community pharmacy pilot UTI service patient experience surveys (S: survey number).

**Theme 1: How patients described the service**
*“Great service provided by the local pharmacist, after two failed attempts to contact my GP from 8 am I was told I was on a call back list for 6 pm. Due to my worsening symptoms I chose to access support from my nearest pharmacy they were professional and very efficient.”* S89
*“Efficient service, …… would not hesitate to recommend this service, thank you very much, I would have been waiting 2 days for a doctors [sic] appointment.”* S124
*“This service is excellent and speeds up the progress of being diagnosed as I normally have to wait weeks to be seen by a doctor. I believe this service to be invaluable to all that use it.”* S14
*“Excellent & thorough service. Far easier than GP surgery.”* S123
*“The service I received was phenomenal!”* S23
**Theme 2: How patients described the pharmacist/staff**
*“The pharmacist at [redacted] pharmacy (I think his name was [redacted]?) went above and beyond and should be commended for his professionalism.”* S23
*“Staff were very professional. The pharmacist took me to a side room to have privacy to discuss my condition.”* S35
*“I am so glad my GP told me to go to the pharmacy, I was seen within 10 min and the whole thing was super friendly and helpful. Pharmacist was so professional, I did not feel embarrassed to talk about it at all.”* S79
*“Absolutely amazing service, just called the pharmacy, they gave me a same day appointment and I was dealt with in a private and professional manner, the pharmacist explained everything very clearly and was precise. An excitement [sic] service which I highly recommend.”* S142
*“Pharmacist explained why I don’t need antibiotics today, thanks for reassuring me”* S51
**Theme 3: How patients described their feelings**
*“The pharmacist was super friendly and super helpful and I felt really supported.”* S26
*“This is a wonderful service. I felt empowered. Resolving this problem and receiving advice from Pharmacist on why my symptoms were not a UTI allowed me to get an appointment with my GP to follow up my symptoms. Thank you” S18*
*“Really grateful for today, thanks!”* S43
*“I thought the service was excellent. I had taken a urine sample to the doctors [sic] surgery, but it wouldn’t be done until later that day. It was a Friday and I was going away that weekend. I was really uncomfortable and in pain. Seeing the pharmacist helped a lot and having the antibiotics made my weekend a lot more comfortable. When I was given antibiotics and listened to, I cried with relief. I would definitely recommend and would use this service again. Knowing that this is available when the doctors [sic] surgery is closed, is a relief. Thank you for providing this service. Please continue with this service as I will recommend it to others.”* S71

## Data Availability

The data presented in this study are available on request from the corresponding author. The data are not publicly available due to privacy.

## Supplementary Materials

The following supporting information can be downloaded at: https://www.mdpi.com/article/10.3390/antibiotics14111086/s1, Figure S1: A flowchart summarising the pathway for managing diagnosis of a urinary tract infection, as per the All Wales Common Ailments Service formulary; Table S1: Responses to questions in relation to patient-reported experiences in the survey (n = 309); Table S2: Antibiotic supply and demographic characteristics of patients in the sample in the current study (n = 309) and the total population cohort who had a UTI consultation in the pharmacy between 17 June 2024 and 31 January 2025 (n = 9077).
