# Supplementary material for: “I Felt Empowered”: Patient-Reported Experience with a Pilot National Community Pharmacy-Based Urinary Tract Infection Service
Source: Antibiotics (Basel). 2025 Oct 28;14(11):1086. doi: 10.3390/antibiotics14111086 (PMC12649495; doi:10.3390/antibiotics14111086)
Supplement: Supplementary file 1 [file antibiotics-14-01086-s001.zip › antibiotics-3948033-supplementary.pdf]

Supplementary tables/figures

Figure S1: A flowchart summarising the pathway for managing diagnosis of a urinary tract infection, as per the All Wales Common Ailments Service formulary

(https://awttc.nhs.wales/medicines-optimisation-and-safety/medicines-optimisation-guidance-resources-and-data/prescribing-guidance/all-wales-common-ailments-service-formulary/ page 131)

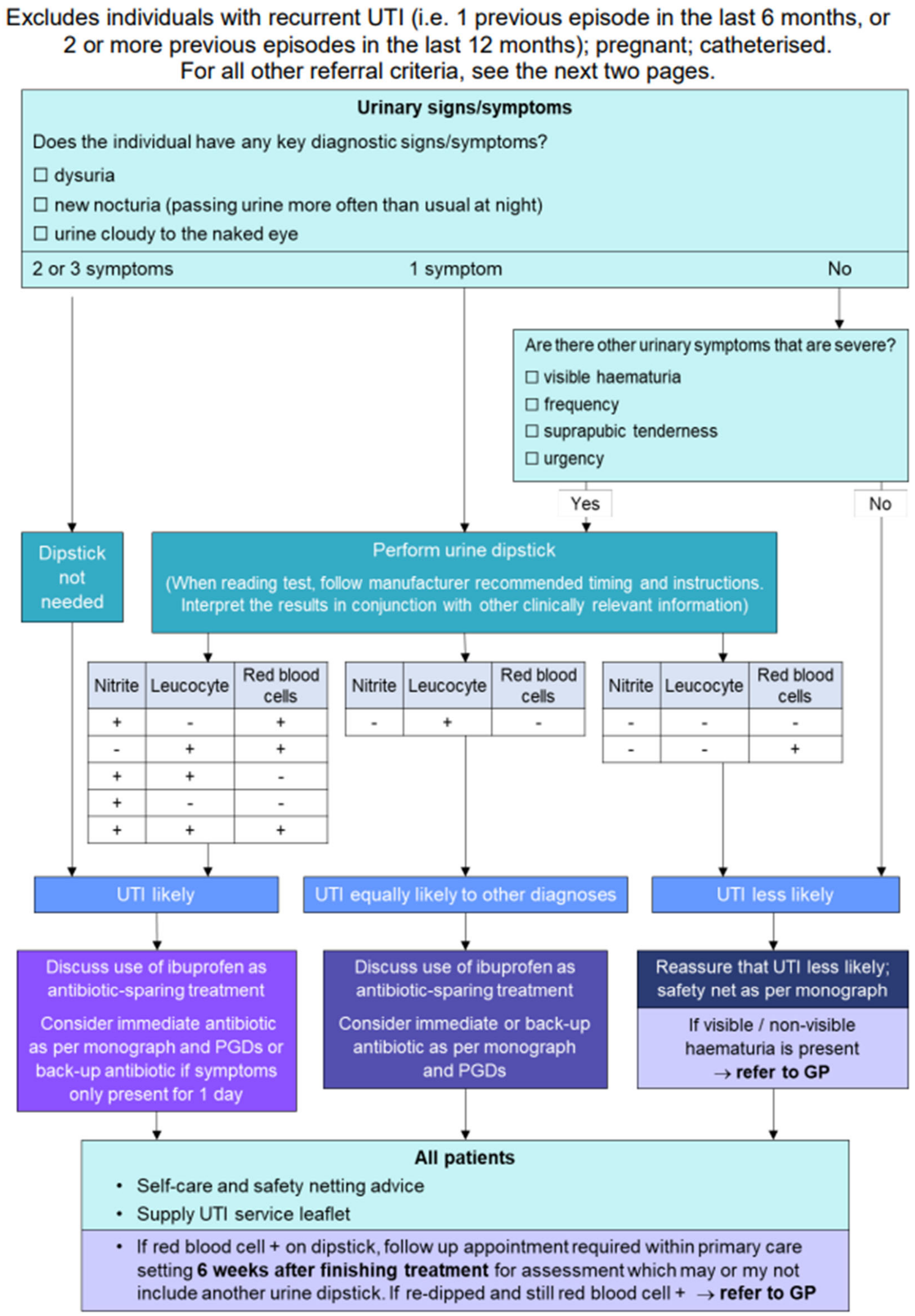

Table S1: Responses to questions in relation to patient-reported experiences in the survey (n=309).

	**Strongly disagree (N (%))**	**Disagree (N (%))**	**Neither agree nor disagree (N (%))**	**Agree (N (%))**	**Strongly agree (N (%))**	**Missing/ NA (N (%))**
**Thinking back to the UTI service consultation, what is your level of agreement with the statements below? If any of the statements do not apply to you, please select N/A**						
3.1 The pharmacist explained the urinary tract infection service to me in a way that I could understand	3 (1.0)	1 (0.3)	0 (0.0)	35 (11.3)	270 (87.4)	0 (0.0)
3.2 I was involved as much as I wanted to be in decisions made about my care	2 (0.6)	0 (0.0)	1 (0.3)	42 (13.6)	264 (85.4)	0 (0.0)
3.3 I had the opportunity to ask questions or raise concerns related to the service	2 (0.6)	0 (0.0)	1 (0.3)	27 (8.7)	277 (89.6)	2 (0.6)
3.4 I felt that I was listened to	2 (0.6)	0 (0.0)	0 (0.0)	33 (10.7)	273 (88.3)	1 (0.3)
3.5 If a urine dip stick test WAS NOT needed, the pharmacist explained the reasons why[a]	0 (0.0)	9 (2.9)	8 (2.6)	15 (4.9)	123 (39.8)	154 (49.9)
If a urine dip stick test WAS needed….[a]						
3.6 The pharmacist explained the reasons why	2 (0.6)	2 (0.6)	3 (1.0)	18 (5.8)	164 (53.1)	120 (38.8)
3.7 I am satisfied with the way the pharmacist explained how it would be performed	2 (0.6)	1 (0.3)	2 (0.6)	17 (5.5)	157 (50.8)	130 (42.1)
3.8 The results of the test helped me understand if it is likely I have a urinary tract infection	1 (0.3)	1 (0.3)	3 (1.0)	17 (5.5)	162 (52.4)	125 (40.4)
3.9 I am satisfied with the advice the pharmacist provided on how I can manage my symptoms after I leave the pharmacy	1 (0.3)	1 (0.3)	2 (0.6)	26 (8.4)	278 (90.0)	1 (0.3)
3.10 I am satisfied with the way the pharmacist explained what to do if my current symptoms get worse	1 (0.3)	0 (0.0)	3 (1.0)	21 (6.8)	280 (90.6)	4 (1.6)
After speaking to the pharmacist:						
3.11 I understand why antibiotics are not always recommended	1 (0.3)	4 (1.3)	15 (4.9)	36 (11.7)	217 (70.2)	36 (11.6)

3.12 I feel more confident about managing my current symptoms	1 (0.3)	0 (0.0)	1 (0.3)	36 (11.7)	265 (85.8)	6 (1.9)
3.13 I understand how I can try to prevent getting urinary tract infections in the future	1 (0.3)	8 (2.6)	23 (7.4)	42 (13.6)	218 (70.6)	17 (5.5)
**Thinking about your future actions, what is your level of agreement with the statements below?**						
	**Strongly disagree**	**Disagree**	**Neither agree nor disagree**	**Agree**	**Strongly agree**	**Missing/ NA**
5.1 I would recommend the pharmacy urinary tract infection service to others	1 (0.3)	0 (0.0)	0 (0.0)	24 (7.8)	284 (91.9)	0 (0.0)
5.2 Next time I think I have a urinary tract infection, I will return to the pharmacy instead of trying to see a GP	2 (0.6)	1 (0.3)	9 (2.9)	27 (8.7)	270 (87.4)	0 (0.0)

[a] Patients responding to where a urine dip stick test was and was not needed do not add up to N=309 .i.e. some patients responded to both questions, and some responded to neither.

Table S2: Antibiotic supply and demographic characteristics of patients in the sample in the current study (n=309) and the total population cohort who had a UTI consultation in the pharmacy between 17th June 2024 and 31st January 2025 (n=9,077).

	**Sample of patients completing the survey (n=309) (%)**	**Total population cohort between 17th June 2024 and 31st January 2025 (n=9,077) (%)**
Antibiotic supply	75.7	78.7
Age		
16-17	1.9	2.8
18-24	22.7	15.2
25-34	18.8	21.4
35-44	20.4	21.6
45-54	20.4	18.7
55-64	15.9	20.3
